# Evaluating genetic drift in time-series evolutionary analysis

**DOI:** 10.1016/j.jtbi.2017.09.021

**Published:** 2018-01-21

**Authors:** Nuno R. Nené, Ville Mustonen, Christopher J. R. Illingworth

**Affiliations:** a Department of Genetics, University of Cambridge, Cambridge, UK; b Wellcome Trust Sanger Institute, Wellcome Trust Genome Campus, Hinxton, Cambridge CB10 1SA, UK; c Department of Biosciences, Department of Computer Science, Institute of Biotechnology, University of Helsinki, Helsinki 00014, Finland

**Keywords:** Genetic drift, Time-resolved genome sequence data, Wright–Fisher model, Experimental evolution

## Abstract

The Wright–Fisher model is the most popular population model for describing the behaviour of evolutionary systems with a finite population size. Approximations have commonly been used but the model itself has rarely been tested against time-resolved genomic data. Here, we evaluate the extent to which it can be inferred as the correct model under a likelihood framework. Given genome-wide data from an evolutionary experiment, we validate the Wright–Fisher drift model as the better option for describing evolutionary trajectories in a finite population. This was found by evaluating its performance against a Gaussian model of allele frequency propagation. However, we note a range of circumstances under which standard Wright–Fisher drift cannot be correctly identified.

## Highlights

• We assess the inferrability of a Wright–Fisher drift model from time-resolved genome sequence data.
• We identify thresholds at which a Wright–Fisher model can be distinguished from Gaussian diffusion.
• Considering a recent experimental dataset, a Wright–Fisher model is favoured.
• We infer chromosome dependent effective population sizes for this dataset.

## 1 Introduction

Rapid advances in high-throughput methodologies have enabled the collection of rich time-series from experimental evolution studies. These typically address the effects of environmental conditions on adaptation stemming from *de novo* mutations (Barrick and Lenski, 2013), initial variance induced by a genetic cross (Bergström, Simpson, Salinas, Barr, Parts, Zia, Nguyen Ba, Moses, Louis, Mustonen, Warringer, Durbin, Liti, 2014, Culleton, Martinelli, Hunt, Carter, 2005, Mancera, Bourgon, Brozzi, Huber, Steinmetz, 2008) or simply from the standing variation characterizing a polymorphic starting population (Schlötterer et al., 2014). Sequencing the emerging populations during these types of experiments allows for identification of molecular aspects behind the species’ reproductive success.

Despite advances in the field, a challenge remains regarding the optimal approach for identifying loci under selection given time-resolved genomic data. Due to linkage disequilibrium, selection at a single locus can lead to changes in allele frequencies across multiple loci (Hill and Robertson, 1966), confounding single-locus approaches to the inference of selection (Illingworth and Mustonen, 2011). Further, in smaller populations, genetic drift may have a significant impact upon allele frequencies, such that the influence of selection must be distinguished from stochastic effects, arising from both propagation and sampling (Charlesworth, 2009, Jónás, Taus, Kosiol, Schlötterer, Futschik, 2016, Jorde, Ryman, 2007).

A variety of methods have been proposed for inferring selection in time-series under genetic drift, utilising the Wright–Fisher drift model for forward propagation (Ewens, 2012), approximations to the Wright–Fisher model (Feder, Kryazhimskiy, Plotkin, 2014, Lacerda, Seoighe, 2014, Tataru, Bataillon, Hobolth, 2015, Terhorst, Schltterer, Song, 2015, Topa, Jónás, Kofler, Kosiol, Honkela, 2015, Waxman, 2011), its diffusion limit (Bollback et al., 2008) and respective spectral decomposition approaches (Song, Steinrücken, 2012, Steinrücken, Bhaskar, Song, 2014), or effective simulation methods (Foll, Shim, Jensen, 2015, Malaspinas, 2016). Recently, an accurate beta approximation has also be shown to model important features at the absorbing boundaries which, otherwise, would not be easily attainable (Tataru et al., 2015) (see also Tataru et al. (2016) for an extensive review of other methods). However, while the Wright–Fisher model has become the standard approach to representing genetic drift, it is built upon certain modelling assumptions, including the replacement of the entire population in successive generations. As such, other models may in some respects provide a better fit to the dynamics observed in evolutionary experiments (Der et al., 2011). Experimental demonstrations intended to validate the Wright–Fisher model have suffered from limitations in the extent of data available for analysis (Buri, 1956, Der, Epstein, Plotkin, 2011).

Here, we evaluate the extent to which a Wright–Fisher model of genetic drift can be inferred from data pertaining to evolutionary trajectories, contrasting it with a model of Gaussian diffusion. The Gaussian model at first sight differs greatly from the Wright–Fisher model, lacking frequency-dependent variance, albeit we note that, when compounded with the effect of finite sampling, frequency-dependent variance does arise in the Gaussian model. A further contrast is noted in the computational efficiency of the algorithms; the Gaussian model is analytically solvable, allowing for rapid evaluation, whereas the Wright–Fisher model is more computationally intensive. We test the extent to which a model of drift is identifiable from simulated allele frequency data and a large dataset from evolutionary experiments conducted in *Drosophila melanogaster* (Franssen, Nolte, Tobler, Schlötterer, 2015, Orozco-terWengel, Kapun, Nolte, Kofler, Flatt, Schlötterer, 2012). We note that correct inference of a Wright–Fisher model is not always possible from simulated Wright–Fisher data, with various parameters influencing model identifiability. However, data from evolutionary experiments shows evidence in favour of a Wright–Fisher drift model under a likelihood-based inference approach.

## 2 Results

The potential to correctly identify a model of drift was evaluated using a Hidden Markov Model with an independent emission component, based on a version of the Kalman filter (Barber, 2012, Fischer, Vázquez-García, Illingworth, Mustonen, 2014). In general terms, we represented the frequency of an allele as a probability distribution, propagated at each generation, and observed via a finite sequencing process. Our emission model thus represents a form of uncertainty equivalent to that arising from evolutionary experiments that have used the Pool-Seq paradigm (Kofler et al., 2012). Given Gaussian and Wright–Fisher models of propagation, their relative fit to the data was evaluated using a compound log-likelihood difference, with optimal parameters identified by a standard non-linear optimization technique.

**Fig. 1:**
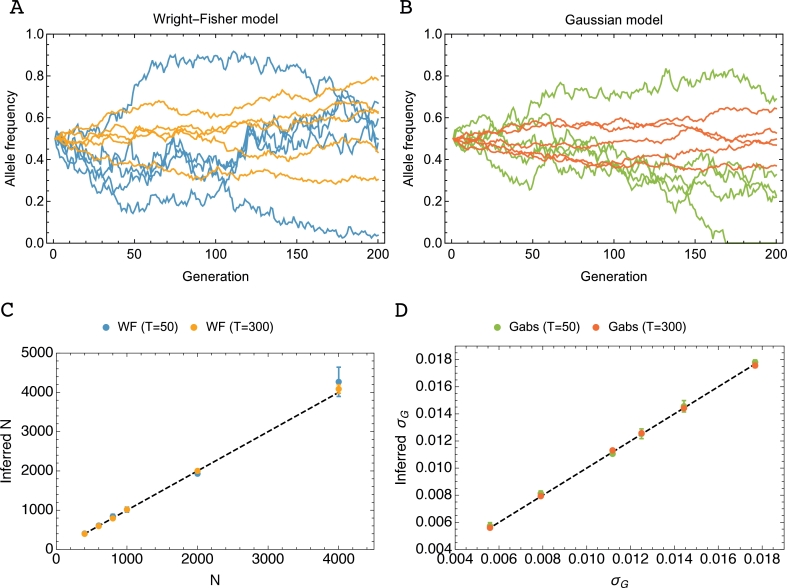
Wright–Fisher and Gaussian models of allele frequency propagation and accuracy in drift parameter inference. (A) Example trajectories generated under a Wright–Fisher model with population sizes N=400 (blue) and N=4000 (yellow). (B) Example trajectories generated under a model of Gaussian diffusion with $\sigma_{G}=0.018$ (green) and $\sigma_{G}=0.006$ (red). (C) Inferred versus simulated population sizes given observations over T=50 and T=300 generations of simulated data generated with exact Wright–Fisher propagation. (D) Inferred *σ_G_* vs simulated *σ_G_* for equivalent calculations using the Gaussian model for trajectories. Simulations used for inference were generated with read depth C=100, sampling period Δt=10, and starting frequency q(0)=0.5. (For interpretation of the references to colour in this figure legend, the reader is referred to the web version of this article.)

In order to test our ability to infer correct parameters from simulated data, given the combination of the drift model with an emission component, we tested our model against 2 batches of simulations covering several population sizes and variances for the Wright–Fisher and the Gaussian model respectively. Fig. 1, shows that accurate parameter inference was achieved under each drift model. At large population sizes (or smaller variances), the expected rate of change in an allele frequency declines, so that a longer period of observation, represented by *T*, the trajectory length, was required to estimate *N* (or *σ_G_*) to a high level of accuracy. Given 300 generations of data, accurate estimates of *N* or *σ_G_* were obtained from all simulated populations (see Supporting Text for consideration of the effect of the number of trajectories on inferred parameters).

**Fig. 2:**
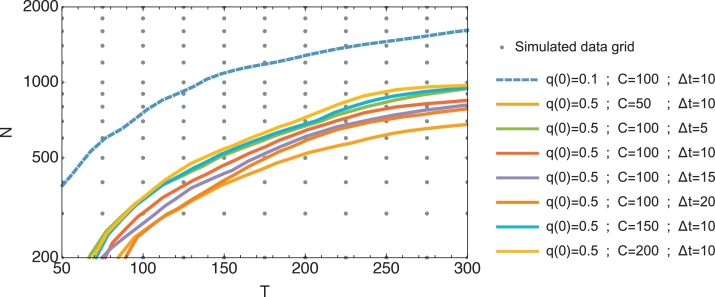
Potential to identify a Wright–Fisher model of evolution. Contours show lines of constant likelihood difference *ΔL* per locus per sampling instant by population size *N* and experimental duration *T*, between the exact Wright–Fisher and Gaussian drift models, when data is generated by Wright–Fisher propagation. Each contour represents the threshold below which correct model identification is possible at comparable likelihood differences. Solid lines show the contour ΔL=0.01; a dashed line shows the contour ΔL=0.05, for each set of parameters. Contours were found by interpolation of data generated at specific combinations of population size and experimental duration, shown as gray dots, and smoothing with an exponential moving average. Log scale is used on the y-axis.

Given sufficient data generated from a pure Wright–Fisher or Gaussian model of drift, correct identification of the drift model could be achieved. However, a threshold time, sometimes of 300 generations or more, was required for this to be achieved (Fig. 2). We tested a diverse set of simulated data with several representative parameters of typical E&R experiments (Kofler and Schltterer, 2014): sequencing depth, sampling period, initial allele frequency, experimental duration and population size. The underlying population size of the system, *N*, was a critical factor in determining the threshold for identification; at higher *N*, the change via drift may be insufficient for model discrimination. Further factors influenced this value; for example, trajectories starting at lower frequencies were more informative of the drift model due to increased frequency dependence, reflected, for example, in the derivative of the characteristic variance. At frequency values closer to the boundaries, q(t)=0 and q(t)=1, the importance of higher-order moments characterizing the Wright–Fisher model are also a strong contributing factor. An increased depth and frequency of sampling increased the extent of information available for inference; each improved the ability for model discrimination (see Fig. 2 and additional results in Supporting Text).

While the simulations discussed above consider systems in which drift is the only force driving evolution, in a biological system, other factors affect allele frequency change. Selection, mutation, and linkage disequilibrium each influence the shape of the expected distribution of allele frequencies with time, potentially affecting the identifiability of a model of drift

Natural selection acting upon a population induces changes in allele frequency over time. As such, including selection in our simulations led to an increased allele frequency variance in our simulation data. Subsequent inference of *N* under a neutral assumption led to underestimates of *N* proportionate to the number of loci at which selection acted. However, the correct inference of a Wright–Fisher drift model in each case was not compromised (see Supporting Information).

The rate of mutation in experimental systems relevant to our work, of close to $\mu \approx 10^{-9}$ (Li and Stephan, 2006), has an influence on allele frequencies much smaller than the effect of genetic drift. To explore the theoretical effect of mutation, simulations were conducted with much higher rates of mutation. From simulated data, population sizes were over-estimated if the starting frequency was 0.1 and μN=0.1 or 0.5, and under-estimated if μN=1 or 10 (see also Supplementary Information). At low frequencies, the influence of mutation led to incorrect model identification; the Gaussian distribution describes with greater flexibility the sample paths generated by the balance between drift, which pushes trajectories towards either of the absorbing boundaries, and mutation, which drives the frequency spectrum away from a frequency of 0 or 1. Where *μN* is sufficiently high, drift is overcome by the tendency of mutation to push frequencies to q(t)=0.5. Considering simulations with a starting frequency of 0.5, consistent overestimates of *N* were obtained to compensate for the effect of mutation keeping the allele frequency close to a constant value. However, in these cases, the Wright–Fisher model was correctly identified in comparison to the Gaussian drift model.

The presence of linkage disequilibrium between loci may act as a confounding factor for selection identification. Yet, for model identifiability without selection, hitch-hiking effects should only have a significant impact if the number of founding haplotypes is reduced or if the size of genomes is small (Franssen, Nolte, Tobler, Schlötterer, 2015, Terhorst, Schltterer, Song, 2015). Under these conditions, a random bias in allele frequency change may be observed, leading to possible incorrect model identification. For the simulated genomes under a neutral coalescent model employed here (see Methods), propagation with linkage, even for a low number of founding haplotypes, did not lead to incorrect drift model identification. Population sizes for these datasets were slightly over-estimated (see Supplementary Information).

**Fig. 3:**
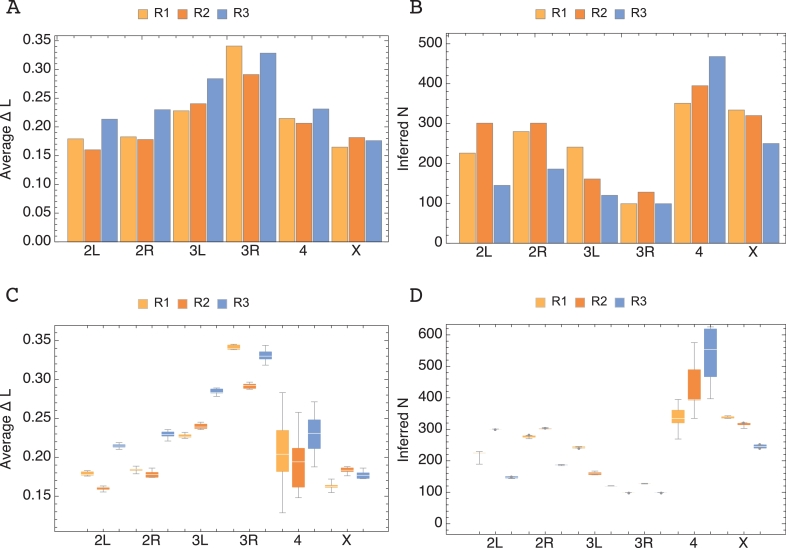
Population size estimates from Drosophila experimental evolution time-series (Franssen et al., 2015) and average likelihood per locus, between exact Wright–Fisher and Gaussian propagation with absorbing boundaries. R1, R2, R3 represent estimates from different experimental replicates reported in Franssen et al. (2015) (see Methods for further details). Boxplots in (C) and (D) correspond to the Average *ΔL* per locus and respective population size estimates for sets generated by bootstrapping (see Methods).

Applying the model to experimental genomic data (Franssen et al., 2015), an improved fit was not seen for the Wright–Fisher model across all statistical measures considered (see Supporting Text, where the error in the estimated compound variance is evaluated). However, a clear result in favour of this model was seen via a likelihood calculation. Estimated population sizes calculated under the Wright–Fisher model are shown in Fig. 3 (A). Consistent with the identification of selection in the data (Franssen et al., 2015), these estimates are lower than the reported consensus size of 1000. Further calculations were performed to evaluate models of drift over the subset of loci in all chromosomes that did not reach fixation. This was intended to verify whether the improved performance of the Wright–Fisher model arose from the natural inclusion of fixation events in this drift model; a more artificial approach was required in the case of the Gaussian drift model. While average likelihood differences for this dataset were reduced, the tendency across chromosomes observed in Fig. 3 was not altered.

In the results of Fig. 3, differences between the estimates obtained were observed for different replica datasets. As noted in supplementary Fig. F.14, the differences between initial distributions is minimal, likely excluding this as an explanation for the differences. In order to estimate the variance in our estimates, a bootstrapping procedure was applied, examining sets of trajectories from *L*/10 loci in each case, where *L* is the number of loci in the original chromosomes with identified variant alleles; 100 bootstrapping sets per chromosome and replicate were performed. As can be seen in Fig. 3 (C) and (D), even under this conservative procedure little variance in estimates was observed, with the exception of the small chromosome, 4. Intrinsic differences between replicates may underlie the different results (Franssen et al., 2015).

## 3 Discussion

The Wright–Fisher model is the most popular discrete-time approach for modelling populations, describing their fine-structure as the result of a succession of randomly drawn, non-overlapping sampling generations at a constant consensus size. However, evaluation of the explicit model is computationally intensive, requiring repeated matrix multiplications. For this reason, published approaches for inferring selection within a population of finite size have utilised a variety of approximations to the Wright–Fisher model when accounting for genetic drift.

Here, we have considered the extent to which a Wright–Fisher model is possible to infer from time-resolved allele frequency data. Applied to a large dataset from an evolutionary experiment, we demonstrate that it is identifiable under a likelihood model. In so far as a drift model can be compared to arbitrarily similar models, it can never truly be proven to be correct through the analysis of experimental data. Nevertheless, under the approach outlined here, we have identified a Wright–Fisher model of genetic drift as outperforming a model of drift via Gaussian noise when applied to data from a biological population.

Our calculations on simulated data further show that the identification of Wright–Fisher drift is not trivial, and may not be replicable in other datasets; in situations where the time over which a population is observed is short, where the underlying population size is large, or where sampling is shallow or sparse, Wright–Fisher drift may be indistinguishable from variance in a Gaussian model. Under such circumstances the potential for the use of alternative, rapid approximations to the Wright–Fisher approach is clear. The Gaussian model described here provides one such approach, for which an analytical solution is possible; scope remains for research into fast and flexible alternative procedures applicable to situations where data is scarce, intricate parametric approaches are not possible or population models are not identifiable. Under these circumstances, Bayesian non-parametric inference frameworks may stand as a viable option for evolutionary time-series analysis (Orbanz, Teh, 2010, Topa, Jónás, Kofler, Kosiol, Honkela, 2015).

Experimental evolution and the analysis of the resulting time-series data have provided extensive proof for a number of evolutionary modes responsible for overall trends leading to particular phenotypic outcomes (Barrick, Lenski, 2013, Schlötterer, Tobler, Kofler, Nolte, 2014). Several simulation studies have also tested the success of a number of typical experimental set-ups in providing information on loci responding to environmental stress (Kofler and Schltterer, 2014), the present paper having a specific focus on stochasticity in evolutionary analysis. Optimising the design of experiments so as to maximise the information obtained (Liepe et al., 2013) may be an important step in validating Wright–Fisher population models (Der et al., 2011), or distinguishing between potential approximations (Tataru et al., 2016). Further investigations are necessary to systematically quantify the utility of certain experimental designs; a decision toolkit based on the mutual information between experiment and theoretical framework could improve our understanding of the potential to predict and control evolution (Lässig et al., 2017).

## 4 Methods

### 4.1 Simulated data generation

Simulations were performed using an exact Wright–Fisher model. Parameters for simulations were chosen to reflect those relevant to recent Evolve and Resequence (E&R) experiments (Franssen, Nolte, Tobler, Schlötterer, 2015, Kofler, Schltterer, 2014, Orozco-terWengel, Kapun, Nolte, Kofler, Flatt, Schlötterer, 2012) and representative simulation studies (Kofler and Schltterer, 2014), including the population size (*N*), initial frequency distribution (*q*(0)), sequencing coverage depth (*C*), experiment length (*T*), sampling period (*Δt*), number of replicates and number of loci (*L*) used to infer population parameters.

In order to test the accuracy of our inference method an additional test was performed with simulations generated by a Gaussian diffusion model on the interval [0, 1] with absorbing boundaries (inference results in Fig. 1 and Supporting Text).

In order to evaluate drift model identification under the inference framework outlined below, two batches of Wright–Fisher simulations were studied. One considered evolution at a single locus, where trajectories were completely independent. For this batch, we tested model identification on both trajectories with and without mutation. A Poisson model was used when mutation was present. As with the neutral trajectories without mutation, several population sizes were used in the interval [100, 5000]. For each population size several mutation rates were analysed in order to cover the region of *μN* values where the frequency spectrum changes its shape; the selected set for *μN* was {0.1, 0.5, 1, 10}. From μN=10 to μN=0.1, a transition occurs where around μN=0.5 the system goes from having the most probable value located at q(t)=0.5 to having two significant spikes at the absorbing boundaries (Rouzine et al., 2001). An additional subset of simulations was generated to study the effects of selection on inference. These included selection coefficients sampled from a uniform distribution in the interval [−0.01,0.01], for either 1% or 10% of the loci.

The second batch of Wright–Fisher simulations was based on propagation of genomes with linkage characteristic of *Drosophila melanogaster* (100 cases in total). The program *FastSimCoal* (Excoffier et al., 2013), under a neutral coalescent model, was used to generate the starting genomes, with roughly L=5000 polymorphic positions; 2000 sequences were used in this instance. The propagation of populations under Wright–Fisher dynamics, at a constant census size, for genomes of length *L*, was performed by a set of purpose built routines (see Section 5). In order to construct the starting population we sampled 2*N*/*F* times from the set of haplotypes generated by *FastSimCoal*, with F=20 representing the number of founding sequences. Further simulations with a higher number of haplotypes could have been tested. Yet, it was verified that even for a low number of starting genomes the task of correct model identification was not hindered; higher *F* should not change considerably the results. Lower *F*, on the other hand, may lead to spurious effects at the allele frequency level due to linkage (Terhorst et al., 2015). The mutation rate (*μ*) and the recombination rate (*ρ*) for the coalescent neutral model were imposed at $\mu =3\times 10^{-9}/bp/gen$ and $\rho =10^{-8}/bp/gen,$ consistently with the experimentally determined recombination rates and the recombination rate calculator (Comeron, Ratnappan, Bailin, 2012, Fiston-Lavier, Singh, Lipatov, Petrov, 2010). For both batches of simulations a binomial sampling process was used to simulate sequencing of the population (see Eq. (1)).

As we are studying identification of drift model in evolutionary time-series characteristic of E&R experiments, measured by Pool-Seq, the effects of migration were not addressed. Its contribution to the variance under the one-locus Wright–Fisher neutral model can be studied efficiently through standard methods for recursive discrete dynamical systems (Tataru, Bataillon, Hobolth, 2015, Tataru, Simonsen, Bataillon, Hobolth, 2016). Here, we also do not address recombination during the duration of the experiment. Its effects have been proven to increase the success rate in identification of loci under selection (Kofler and Schltterer, 2014). Since we study linkage disequilibrium in isolation, i.e. no mutation nor selection involved, if the starting point are the genomes generated under a neutral coalescent model, recombination would only allow us to transform the observed frequency dynamics into the one-locus independent case reported in Fig. 2. There, the general limits in drift model identification from evolutionary time-series data are amply shown. In addition, recombination events have been seen to occur rarely in E&R studies in *Drosophila* (Franssen et al., 2015). We also did not study the combined effects of linkage, mutation and selection, since our objective was to isolate the contribution of each of these additional factors to drift model identification. More complicated dynamics are of interest but fall beyond the scope of this work.

### 4.2 Experimental data: temporal allele frequencies determined by Pool-Seq

We analysed the data pertaining to all chromosomes reported in Franssen et al. (2015) and available from *Dryad* (http://datadryad.org) under the accession number *doi*: 10.5061/*dryad*.403*b*2. The experiments performed in Franssen et al. (2015) concerned the adaptation of Drosophila to a novel laboratory environment and are part of an ongoing long term experimental evolution study (see for example Kapun et al. (2014); Orozco-terWengel et al. (2012); Tobler et al. (2014); Versace et al. (2014)). The flies were cultured in a fluctuating temperature and light regime to mimic natural conditions: the new temperature regime was cycled every 12 h between a temperature of 18 and 28 °*C*, which coincided with dark and light periods, respectively. 3 replicates were collected at generations 0, 15 (23 for replicate 2), 37 and 59, and allele frequencies were estimated from Pool-Seq data. The census population throughout the experiment was approximately 1000. For further details on the experimental protocol used to generate the populations at each generation and replicate see the original paper (Franssen et al., 2015). Here, we will focus on identification of drift model from the reported time-series profile. The overall tendency for each chromosome can be seen in the respective frequency probability density functions at each sampling generation reported in Supporting Information. We must emphasize that the method presented here for drift model identification is based on evaluation, under a log likelihood approach, of each locus trajectory given a global drift model parameter, which we find by optimizing the sum of log likelihoods across all positions (see Eq. (4)). Therefore, the probability density profiles presented in Supporting Information are for visual inspection only. Their shape is not taken directly in the inference process, unlike previous studies (Der et al., 2011).

### 4.3 A continuous state-space HMM for integer data

Inferences of drift parameters were conducted using a continuous state-space Hidden Markov Model (HMM) for one-dimensional integer data based on a version of the Kalman filter (Barber, 2012, Fischer, Vázquez-García, Illingworth, Mustonen, 2014). As with traditional approaches involving HMM, it incorporates a dynamical hidden model, $P(q\left(t_{k}\right)|q\left(t_{k-1}\right),\theta )$ and an emission model, *P*(*D*(*t_k_*)|*q*(*t_k_*)), where $D_{i}\left(t_{k}\right)=\left\{n_{i}\left(t_{k}\right),C_{i}\left(t_{k}\right)\right\}$ describes the number of observations of a specific allele *n_i_*(*t_k_*), and the total read depth *C_i_*(*t_k_*), at generation *t_k_* and for each locus *i* in a data set. Here, by default, we assumed that for the pooled population each individual contributed equally, thus leading to a simple binomial emission model, that is:

(1) P(Di(tk)|qi(tk))=(Ci(tk)ni(tk))qi(tk)ni(tk)(1−qi(tk))(Ci(tk)−ni(tk))

Estimation of parameters *θ* was achieved via a forward algorithm, consisting of multiple predict-update steps, by combining sampling with a period $\Delta t=t_{k}-t_{k-1}$ generations and propagation $P(q\left(t_{k}\right)|q\left(t_{k-1}\right),\theta )$:

(2) $$\begin{array}{ccc} & & P(q_{i}\left(t_{k}\right)|D_{i}\left(t_{1:k-1}\right),\theta )\\ & & \;=\;\int dq_{i}\left(t_{k-1}\right)P\left(q_{i}\left(t_{k}\right)|q_{i}\left(t_{k-1}\right),\theta \right)P\left(q_{i}\left(t_{k-1}\right)|D_{i}\left(t_{1:k-1}\right),\theta \right) \end{array}$$

and

(3) $$\begin{array}{ccc} & & P(q_{i}\left(t_{k}\right)|D_{i}\left(t_{1:k}\right),\theta )\\ & & \;=\frac{P\left(D_{i}\left(t_{k}\right)|q_{i}\left(t_{k}\right)\right)P\left(q_{i}\left(t_{k}\right)|D_{i}\left(t_{1:k-1}\right),\theta \right)}{\int dq_{i}\left(t_{k}\right)P\left(D_{i}\left(t_{k}\right)|q_{i}\left(t_{k}\right)\right)P\left(q_{i}\left(t_{k}\right)|D_{i}\left(t_{1:k-1}\right),\theta \right)} \end{array}$$

leading to the likelihood

(4) $$\begin{array}{ccc} & & L(\theta |D)\\ & & \;=\sum_{i=1}^{L}\sum_{k}\log\int dq_{i}\left(t_{k}\right)P\left(D_{i}\left(t_{k}\right)|q_{i}\left(t_{k}\right)\right)P\left(q_{i}\left(t_{k}\right)|D_{i}\left(t_{1:k-1}\right),\theta \right) \end{array}$$

Optimisation of this likelihood gave an estimate of the drift parameter *θ*. As is clear from the likelihood function (Eq. (4)) the full combined algorithm was not necessary to achieve estimates of the drift parameter under each evolutionary model. We, nevertheless, did resort to the weighting scheme underlying the forward-backward/predict-update algorithm in order to generate posteriors for each trajectory, in order to find means and variances characterizing the genomic evolutionary data, and to evaluate model performance under alternative metrics.

We note that, in some cases, Pool-Seq experiments may involve the selection of a subset of individuals from the pool for sequencing. In this case, Eq. (1) may be altered to derive an expression

(5) $$P\left(D_{i}\left(t_{k}\right)|{\hat{q}}_{i}\left(t_{k}\right)\right)P\left({\hat{q}}_{i}\left(t_{k}\right)|q_{i}\left(t_{k}\right)\right)$$

where ${\hat{q}}_{i}\left(t_{k}\right)$ is the frequency of the given allele in the subset of individuals chosen for sequencing, and the first part of the equation is equivalent to that for Eq. (1). In our calculations on experimental data, we note that data for the relevant experiment were collected from 500 female flies at each sampling point (Franssen et al., 2015), giving a total of 1000 genomes in the sequencing pool, such that

(6) P(q^i(tk)|qi(tk))=P(n^i(tk)|qi(tk))=(1000n^i(tk))qi(tk)n^i(tk)(1−qi(tk))(1000−n^i(tk))

where ${\hat{n}}_{i}\left(t_{k}\right)$ is the number of genomes in the sample containing the variant allele. Further details about the method are presented in supplementary information.

#### 4.3.1 Transition matrix construction

Within the above framework, models representing both Gaussian and Wright–Fisher variation were implemented. The transition probability density matrix for the Gaussian drift model, $P(q\left(t_{k+1}\right)|q\left(t_{k}\right),\sigma_{G}),$ representing frequency evolution between sampling instants *t_k_* and $t_{k+1}$ was constructed by using the analytical solution of the Fokker–Planck equation for a system driven purely by noise, that is:

(7) $$\frac{\partial P(q,t)}{\partial t}=\frac{1}{2}\frac{\partial^{2}P\left(q,t\right)}{\partial q^{2}}$$

As the normal distribution is a continuous function in the frequency domain, the features associated with the Wright–Fisher at the boundary, namely absorption, are not represented naturally. In order to add this aspect in the Gaussian transition function, we also include absorbing boundaries according to:

$$\begin{array}{ccc} & & P_{G_{abs}}\left(q\left(t_{k+1}\right)|q\left(t_{k}\right),\sigma_{G}\right)\\ & & \;=\left\{ \begin{array}{c} N(q\left(t_{k+1}\right)-q\left(t_{k}\right)|\sigma \sqrt{\Delta t},q\left(t_{k}\right))\;:Cond.\\ \Pi_{0}\left(t_{k}\right)\;:\;q\left(t_{k}\right)\neq 0,1\;\wedge \;q\left(t_{k+1}\right)=0\\ \Pi_{1}\left(t_{k}\right)\;:\;q\left(t_{k}\right)\neq 0,1\;\wedge \;q\left(t_{k+1}\right)=1\\ 1\;:\;q(t_{k})=0,1 \end{array}\right. \end{array}$$

and

(8) $$\begin{array}{cc} Cond. & =\;q\left(t_{k}\right)\neq 0,1\;\wedge \;q\left(t_{k}\right)-3\sigma \sqrt{\Delta_{k+1}}<q\left(t_{k+1}\right)\\ & \;<q\left(t_{k}\right)+3\sigma \sqrt{\Delta t}\;\wedge \;q\left(t_{k+1}\right)\neq 0,1\\ \Pi_{0}\left(t_{k}\right) & =\int_{q\left(t_{k}\right)-3\sigma \sqrt{\Delta t}}^{0}N\left(q\left(t_{k+1}\right)-q\left(t_{k}\right)|\sigma \sqrt{\Delta t},q\left(t_{k}\right)\right)\\ \Pi_{1}\left(t_{k}\right) & =\int_{1}^{q\left(t_{k}\right)+3\sigma \sqrt{\Delta t}}N\left(q\left(t_{k+1}\right)-q\left(t_{k}\right)|\sigma \sqrt{\Delta t},q\left(t_{k}\right)\right) \end{array}$$

Other approaches based on modelling the behaviour near the absorbing boundaries via beta distributions and spikes (Tataru et al., 2015) have also been proven to be a valid approach; these could also be implemented within the HMM model presented above.

Frequency transitions were modelled on an evenly spaced discrete frequency grid on the interval [0, 1], with resolution $\frac{1}{400}$.

For the exact Wright–Fisher propagation model, $P(q\left(t_{k+1}\right)|q\left(t_{k}\right),N),$ no tractable analytical formulation exists allowing immediate computation at any generation *t_k_* (Ewens, 2012). The exact transition matrix between *t_k_* and $t_{k+1}$ was therefore found by exponentiation of the one-generation 2*N* by 2*N* transition matrix,

(9) $$P\left(q\left(t_{k+1}\right)|q\left(t_{k}\right),N\right)=P{\left(q\left(1\right)|q\left(0\right),N\right)}^{\Delta t}$$

where *P*(*q*(1)|*q*(0), *N*) is defined by

(10) Pi,j(q(1)|q(0),N)=(2N2N×qj(1))qi(0)(2N×qj(1))(1−qi(0))(2N(1−qj(1)))

with i,j=1,…,401. For values of *N* smaller or greater than 400, the inverse distance method was used to interpolate between the nearest points on the discrete binomial distribution.

In the construction of the propagator matrix we do not make any extra assumptions such as a one-step process on the propagation grid as was the case in Malaspinas et al. (2012); this simplification forces the Markov chain, represented in the transition matrix, to be restricted to diagonal and off-diagonal matrix entries $P_{i,i+1}$ and $P_{i,i-1}$ (Van Kampen, 1992). Instead, we calculate the full transition matrix for a specific starting frequency involving all entries.

## 5 Code availability

The code used for matrix exponentiation and likelihood minimization is available at: https://github.com/nunonene/Evaluating-genetic-drift-in-time-series-evolutionary-analysis.

Pre-computed Wright–Fisher transition matrices between sampling instants for population sizes above 1000 and frequency grid size of 400 are also available at the same address. For population sizes below 1000 exponentiation is done during optimization.

The set of routines used for propagation of genomes with linkage disequilibrium characteristic of populations of *Drosophila* and under mutation are also available at the same address.

The program used for generating the sequences with linkage characteristic of *Drosophila* populations was *FastSimCoal* under a neutral coalescent model, available at: http://cmpg.unibe.ch/software/fastsimcoal2/.

## Appendix A Supplementary materials

Supplementary Data S1 Supplementary Raw Research Data. This is open data under the CC BY license http://creativecommons.org/licenses/by/4.0/ Supplementary Data S1
