# Supplementary material for: Evaluating genetic drift in time-series evolutionary analysis
Source: J Theor Biol. 2018 Jan 21;437:51–7. doi: 10.1016/j.jtbi.2017.09.021 (PMC5703635; doi:10.1016/j.jtbi.2017.09.021)
Supplement: Supplementary Data S1 — Supplementary Raw Research Data. This is open data under the CC BY license http://creativecommons.org/licenses/by/4.0/ [file mmc1.pdf]

Supporting Information

Appendix A. Validation of optimization method through log-likelihood differences

The likelihood model outlined in the Methods section was initially validated by testing the relative performance of each of the drift models on simulated data generated by exact Wright-Fisher propagation and standard Gaussian diffusion (Fig. A.1).

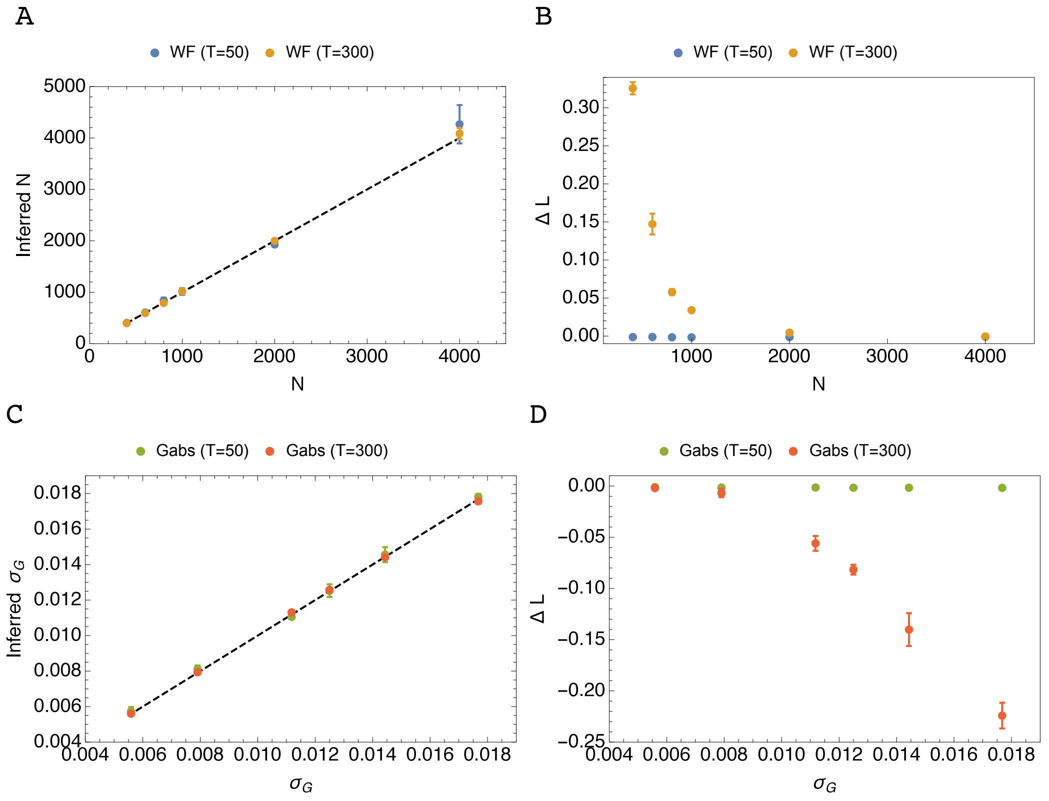

Figure A.1: **Estimates of drift parameter and respective performance with length of evolutionary trajectories (T).** (A) Inferred N and (B) Average relative performance $\Delta L = L_{WF} - L_{G_{abs}}$ per locus when simulated data is generated by traditional Wright-Fisher exact propagation. (C) and (D) equivalent calculations when simulated data is generated by traditional Gaussian propagation. For all figures sequencing coverage depth $C = 100$, sampling period $\Delta t = 10$, grid size 400, starting frequency $q(0) = 0.5$ and number of loci $L = 2000$.

Overall, Fig. A.1 proves that there is no bias in our log-likelihood optimization method; when data is generated with either a Wright-Fisher or a Gaussian model, correct model identification is achieved.

Appendix B. An increased quantity of data improves the inference of population sizes

For the Wright-Fisher model, the dispersion of estimates across replicates is larger for larger population sizes due to poor conditioning at these magnitudes, which arises from the variance characteristic of the Wright-Fisher process being of order $O(\frac{1}{N})$ (see Eq. E.7). This effect and, consequently, the total error in the inferred values decreases with the number of loci used in the estimates (see Fig. B.2).

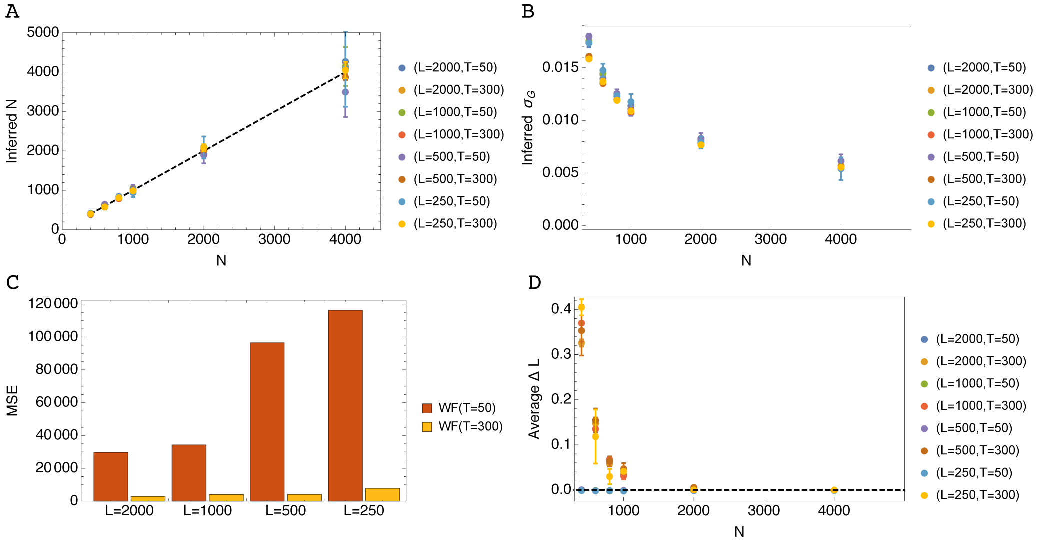

Figure B.2: **Estimates of population size N and respective performance by length of evolutionary trajectories (T), and size of genomes L, for the Wright-Fisher (WF) drift model, when simulated data is generated by traditional Wright-Fisher propagation.** (A) Inferred N and (B) Inferred σ_G vs simulated N for T=50 and 300 generations. (C) Mean-square error between simulated and inferred N. (D) Average performance $\Delta L = L_{WF} - L_{G_{abs}}$ per locus . For all figures sequencing coverage depth $C = 100$, sampling period $\Delta t = 10$, grid size 400 and starting frequency $q(0) = 0.5$. Error bars are calculated across three replicate calculations.

The results reported in Fig. B.2 correspond to simulated data where no selection, mutation or linkage is assumed. Each trajectory is independent.

Appendix C. Sampling factors affecting the correct inference of Wright-Fisher model parameters

Calculations shown in Fig. 2 of the main text were repeated for different values of N, sampling frequency Δt and sampling depth C. In each case model inference was performed for simulated Wright-Fisher trajectories at 2000 loci, of length 300 generations, and starting frequency $q(0) = 0.5$. Greater discrimination between models (observed via an increased likelihood for the Wright-Fisher model) was possible given denser sampling of trajectories, and increased sampling depth, as was also clear from observing the threshold curves' order represented in Fig.2 of the main text. Mean likelihood differences per trajectory and sampling instant are reported in Figure C.3.

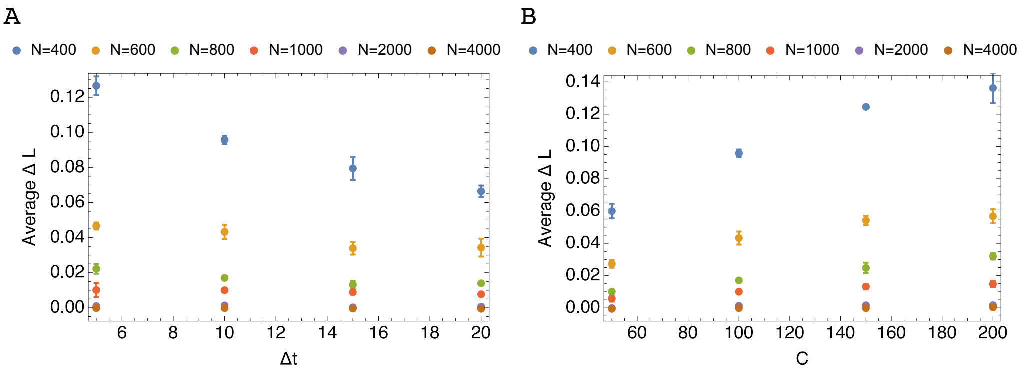

Figure C.3: **Average performance per locus per sampling instant with sampling period (Δt) and sequence coverage depth (C).** (A) Average ΔL by Δt with $C = 100$. (B) Average ΔL by C with sampling period $\Delta t = 10$. For both $T = 300$.

It was expected that trajectory length (T), population size (N) and se-

quencing depth (C) would contribute considerably to model identifiability as these parameters have been previously tested in the context of inference of selection [1, 2]. Sampling period (Δt), on the other hand, has not been as extensively explored in the literature of evolutionary time-series analysis, although the importance of having several time-points in conjunction with replicated trajectories is agreed to be fundamental in order to distinguish between selection and drift in relatively small populations [3]. Recently, it was reported that for Markov chains such as that represented by the Wright-Fisher process, two observations may not determine entirely the behaviour of the stochastic paths at all intermediate instances [4], unless the time between these observations is below a characteristic value. This finding is in close proximity to the importance of sampling frequency determined here and, in addition, to the distribution of sampling instances across the duration of the experiment. Outside evolutionary time-series analysis, the importance of how sparse the collection of information is performed has also been proven to be fundamental in correctly inferring parameters of an underlying diffusion process [5].

Appendix D. Effect of additional evolutionary parameters on drift model identification

Appendix D.1. Natural selection

The presence of selection in the simulated data increases the variance of the observed allele frequencies by introducing a systematic deviation from the mean (see reccurence relations in Eqs. D.1 and D.2) [6]. Yet, it is decreased with respect to the neutral case (Eq. E.7) if each locus is taken independently.

As was mentioned in the Methods section, the simulated data with natural selection was generated with each locus having a random value in the interval $[-0.01, 0.01]$. Under these circumstances, there is a low probability of generating trajectories that, as a group, have a systematic direction towards one of the frequency boundaries. Therefore, if all of the loci are taken together in order to estimate the drift parameter, the observed variance is increased in proportion to the maximum deviation exerted by the combined changes. This additional dispersion in the bulk of trajectories led to an under-estimation of parameters in our neutral likelihood model (Eq. E.1, without the intermediate pool as was used for the experimental data extracted from [7]), with estimates decreasing proportionally with an increase in the number of loci at which selection acted.

$$E_{WF}^{H}[q(t_k)] \approx \frac{(1+s)E_{WF}^{H}[q(t_{k-1})]}{1+sE_{WF}^{H}[q(t_{k-1})]} \tag{D.1}$$

$$\begin{aligned} Var_{WF}^{H}(t_k) \approx& \frac{E_{WF}^{H}[q(t_k)](1-E_{WF}^{H}(q(t_k)))}{2N} \\ &+ \left[\frac{(1+s)}{\left[1+sE_{WF}^{H}[q(t_{k-1})]\right]^2}\right]^2 Var_{WF}^{H}[q(t_{k-1})] \end{aligned} \tag{D.2}$$

As the contribution of selection has a frequency-dependent character (Eqs. D.1 and D.2), correct identification was not compromised under our likelihood model, an advantage in favour of the Wright-Fisher model being inferred in cases where frequency dynamics were observed for sufficient time (Figure D.4). For shorter trajectories, the models tested are indistinguishable.

We must emphasize that we consider selection in the present work as a perturbation to the drift models. For larger selection magnitudes, modelling through a drift-only paradigm is not sufficient; the variance has to be adapted accordingly (see Eqs. D.2) and the relative influence of drift and selection depends on the frequency region a system is at a particular generation [8, 9, 10].

With respect to the compound nature of the likelihood model used throughout our work, we must also highlight that although finite sampling effects led to the Gaussian drift model also having a frequency dependent observed variance (see Eq. E.8, without the intermediate pool), this does not seem to interfere with correct model selection, as higher moments become crucial when the probability density approaches the boundaries, in longer trajectories.

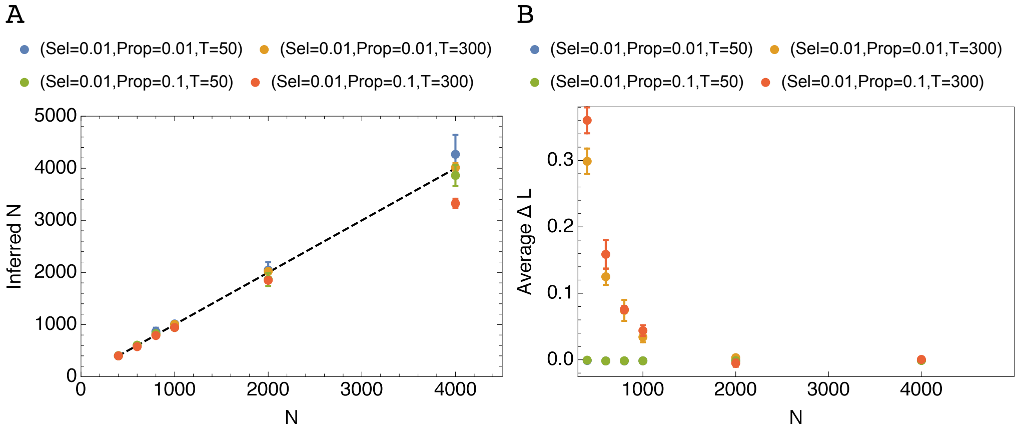

Figure D.4: **Estimates of drift parameter and respective performance with length of evolutionary trajectories (T), when simulated data is generated by traditional Wright-Fisher propagation with selection.** (A) Inferred N vs simulated N, for 300 generations, for several selection strengths and proportion of loci under selection for $q(0) = 0.5$, $C = 100$ and $\Delta t = 10$. (B) Average performance $\Delta L = L_{WF} - L_{G_{abs}}$ per locus corresponding to (A).

Appendix D.2. Mutation

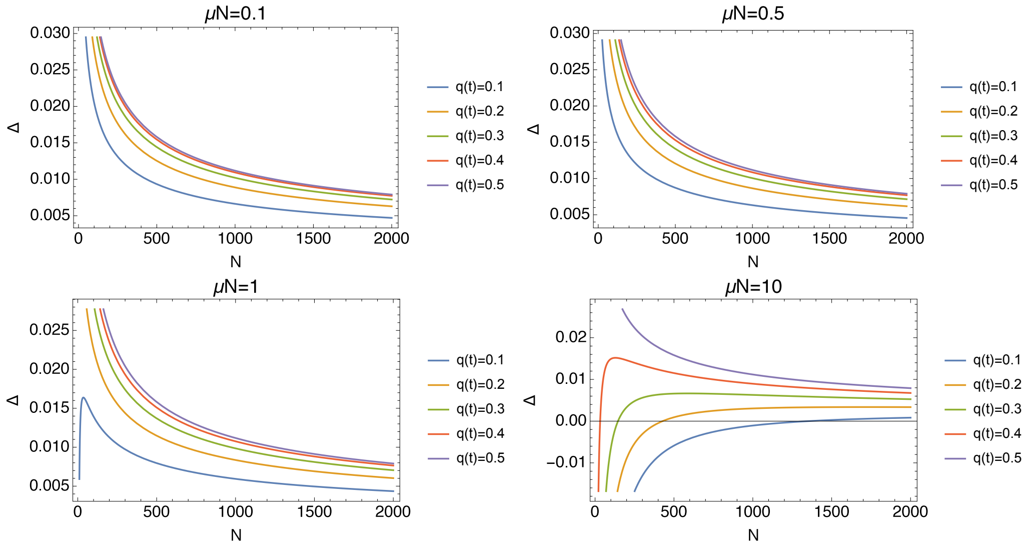

Figure D.5: **Difference in contributions between drift and mutation with population size** N**.** $\Delta = \sqrt{Var^{H}_{WF}(t_k)} - \mid E^{H}_{WF}[q(t_k)] - E^{H}_{WF}[q(t_{k-1})] \mid$ assuming that $Var^{H}_{WF}(t_{k-1}) = 0$. Interpretation of terms according to a diffusion approximation to the Wright-Fisher model with mutation [8].

As we are assuming a one locus case for our drift model, mutation is easily understood by investigating the transition observed for the frequency stationary distributions as μN is changed [8]. Recalling the diffusion approximation to the Wright-Fisher model [8], for values of μN below a threshold in the vicinity of 0.5, a transition ensues where the frequency boundaries occur with high probability and the predicted stationary distribution is roughly U-shaped [8]. Above that threshold the most probable value is $q(t) = 0.5$, at a particular instant t, and the distribution is bell-shaped [8].

In fact, the contributions of drift and mutation to the overall distribution

can also be understood via recurrence relationship for the mean and the variance of the Wright-Fisher process with mutation included, as we did above. For large μN, the contribution of the mutation mostly supersedes the drift term (see Fig. D.5). Only in the region close to $q(0) = 0.5$ is the contribution of the drift term sufficient to overcome the overall tendency imposed by mutation. For lower values of μN, the variance dominates

$$E_{WF}^{H}[q(t_k)] = E_{WF}^{H}[q(t_{k-1})] + \mu \Bigg[1 - 2E_{WF}^{H}[q(t_{k-1})] \Bigg] \tag{D.3}$$

$$\begin{aligned} Var_{WF}^{H}(t_k) = & \frac{E_{WF}^{H}[q(t_k)](1 - E_{WF}^{H}(q(t_k)))}{2N} \\ & + (1-2\mu)^2 \Bigg[1 - \frac{1}{2N} \Bigg] Var_{WF}^{H}[q(t_{k-1})] \end{aligned} \tag{D.4}$$

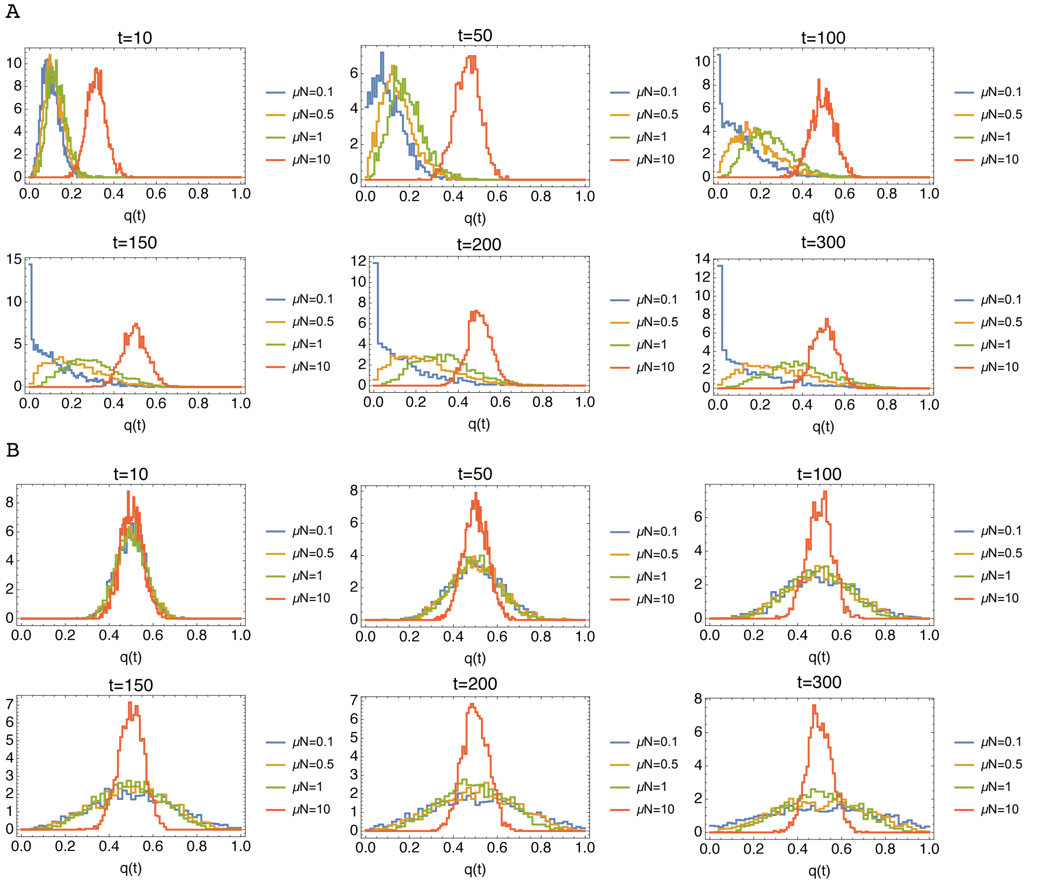

Figure D.6: **Time-dependent hidden probability density from simulated data as a function of μN.** (A) $q(0) = 0.1$. (B) $q(0) = 0.5$. For both $N = 500$.

By propagating populations under mutation throughout the duration of the experiment, we are increasing the proportion of loci distributed around the frequency value $q(t) = 0.5$, either when starting at $q(t) = 0.5$ or $q(t) = 0.1$ (Fig. D.6). Consequently, mutation diminishes the potential for correct model identification in sufficiently long experiments, when the transient initial period is much smaller than the total duration of the experiment. In

fact, for high values of μN, a Gaussian distribution may, in fact, without considering any extra parameters besides drift in our likelihood model (see Eq. E.1), be identified as the best model describing the observed time-series (Fig. D.7). The opposing forces of drift and mutation may be modelled more accurately by a Gaussian model when μN is larger due to the fact that the mutation term in this case is dominant. Also, if the starting frequency is $q(0) = 0.1$, the mutation term indues a systematic movement of the mean towards a frequency of 0.5 which, since the Gaussian model variance is linear with time (see for example Eq. E.5, without the intermediate pool), contributes to its success in modelling the linear character of the general trend enforced by mutation.

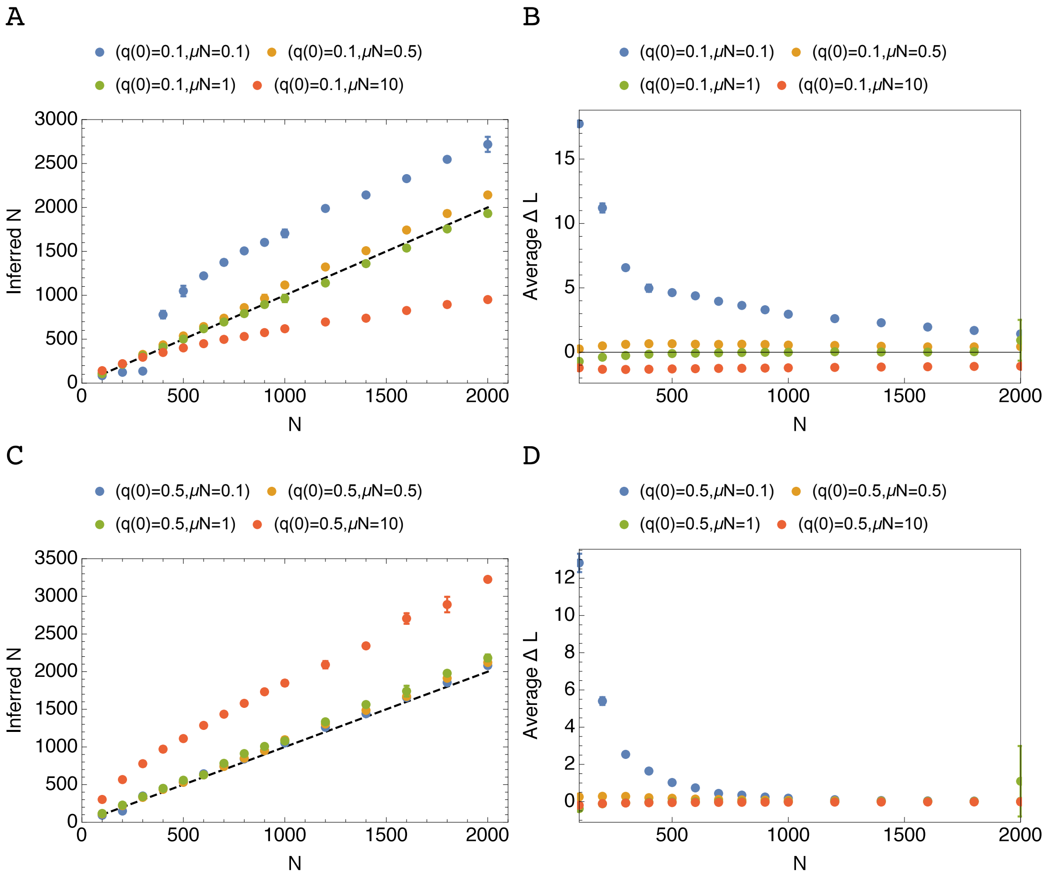

Figure D.7: **Estimates of drift parameter and respective performance with population size (N), when simulated data is generated by traditional Wright-Fisher propagation with mutation.** (A) Inferred N vs simulated N for several mutation strengths measured by μN with $q(0) = 0.1$. (B) Average performance $\Delta L = L_{WF} - L_{G_{abs}}$ per locus corresponding to (A). (C) and (D) similar to (A) and (B), respectively, but for $q(0) = 0.5$. For both $T = 300$, $C = 100$ and $\Delta t = 10$.

Although we tested the influence of μN on drift model inference, mutation rates operating in experimental evolution in *Drosophila* are unlikely to cause any observed mutations to take place during the duration of the experiment. If we consider biologically plausible mutation rates present in $E\&R$ studies,

e.g. $\mu \approx 10^{-9}/bp/gen$ [11], μN will become much smaller than 1, even for populations of order of $\approx 10^6$ [11], leading to the most likely number of mutations appearing at each generation being zero, at least according to a Poisson model. This renders the contribution from mutation terms insignificant (see for example in Fig. D.5 the trend observed from $\mu N = 10$ to $\mu N = 0.1$). Consequently, in this scenario the drift parameter estimates are expected to be slightly over-estimated, if at all.

Appendix D.3. Linkage disequilibrium

Linkage disequilibrium has been proven to be a confounding factor for identification of selection acting on single loci. As such, one might expect the noise signatures characteristic of each of the drift models tested in our work to be muddled. Despite the fact that over-estimation of population size was observed across all cases, correct drift model identification is still achieved (see Fig. D.8).

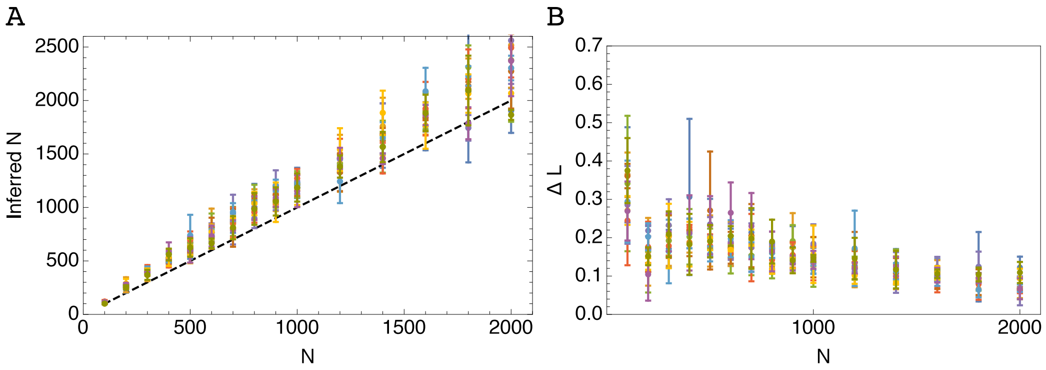

Figure D.8: **Estimates of drift parameter and respective performance with population size (N), when simulated data is generated by traditional Wright-Fisher propagation of founding genomes with linkage disequilibrium characteristic of *Drosophila*.** (A) Inferred N vs simulated N for several mutation strengths measured by μN with $q(0) = 0.1$. (B) Average performance $\Delta L = L_{WF} - L_{G_{abs}}$ per locus corresponding to (A). $T = 300$, $C = 100$ and $\Delta t = 10$. Error bars represent dispersion among replicates. Each color is associated with different examples generated by a coalescent neutral model. See Methods section for details on generated genomes.

Appendix E. Alternative measures for evaluating evolutionary time-series data

Appendix E.1. Combined forward-backward/predict-update posterior and goodness-of-fit calculation

In addition to the computation of the likelihood we also resorted to another statistic, the goodness-of-fit (GOF), taking into account the posterior for each locus frequency at each time-point resulting from the combined forward-backward/predict-update optimization algorithm presented in the Methods section.

As was outlined in the main text, the likelihood function arising from optimization algorithm is

$$\mathcal{L}(\theta|D) = \sum_{i=1}^{L} \sum_{k} \log \int dq_i(t_k) P(D_i(t_k)|q_i(t_k)) P(q_i(t_k)|D_i(t_{1:k-1}), \theta) \quad \text{(E.1)}$$

.

Effectively, $P(q_i(t_k)|D_i(1:t_{k-1}), \theta)$, can be determined in an initial step, referred here as the predict step, where we take the data into account. The emission model $P(D_i(t_k)|q_i(t_k))$ is a compound of binomial distributions as was clarified in the Main Text.

The backward computation is analogous to the forward step described above and the combined forward-backward/predict-update posterior distribution for each locus can be computed by averaging according to Eq. E.2, thus allowing for all of the data to be taken into account, from the initial sampling instant up to the last at $t_k = T$.

$$P(q_i(t_k)|D_i(t_1:T), \theta) = \frac{P(q_i(t_k)|D_i(t_1:t_k))P(q_i(t_k)|D_i(t_{k+1}:T)}{\int dq_i(t_k) P(q_i(t_k)|D_i(t_1:t_k))P(q_i(t_k)|D_i(t_{k+1}:T)} \quad \text{(E.2)}$$

The posterior corresponding to the maximum likelihood estimate can ultimately be used to calculate an additional statistic commonly referred to as Goodness-of-Fit (GOF), see Eq. E.3.

$$\mathcal{GOF}(\theta|D) = \sum_{i=1}^{L} \sum_{k} log \int dq_i(t_k)(q_i(t_k) - q_i^D(t_k))^2 P(q_i(t_k)|D_i(t_1:T), \theta) \quad \text{(E.3)}$$

Eq. E.3 allows us to compute the error, across all loci and sampling instants, in the position of the posterior distribution with respect to the actual data.

Appendix E.2. Drift model identification according to goodness-of-fit applied to experimental data

In agreement with the likelihood calculation of the main text, GOF statistics calculated for the experimental data showed a closer fit to the data for the Wright-Fisher, as opposed to the Gaussian model (Figure E.9).

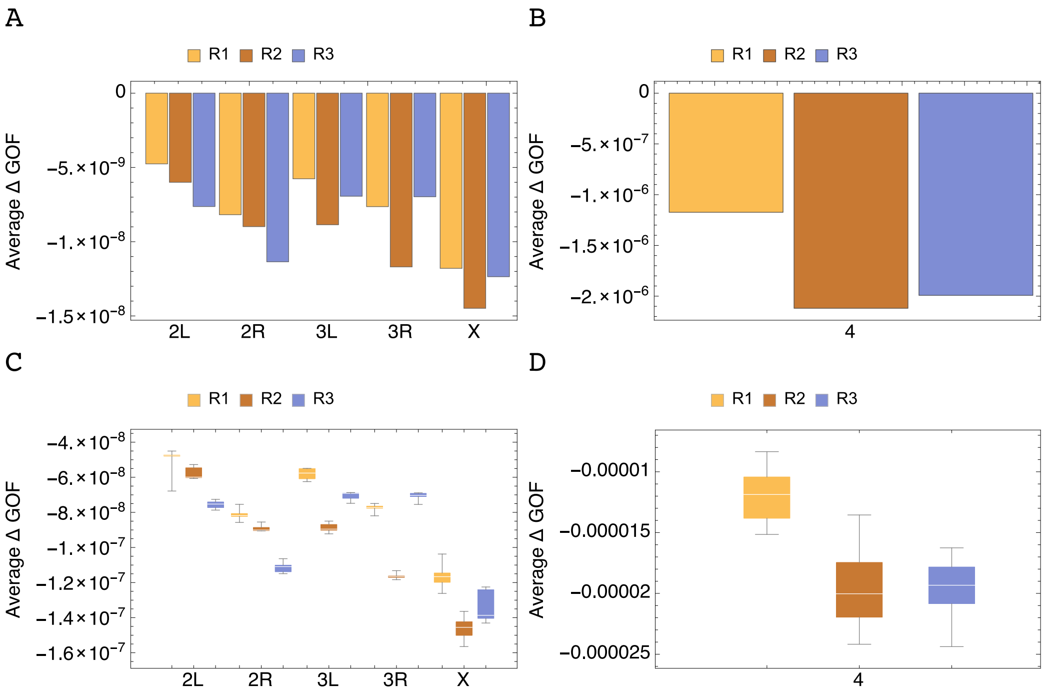

Figure E.9: **Goodness-of-fit difference per locus between exact Wright-Fisher and Gaussian propagation models applied to each each replicate of each chromosome reported in [7].** (C) and (D) correspond to the results on samples generated by bootstrapping (see Methods in Main Text).

Appendix E.3. Estimation of variance across the frequency spectrum

We note that, given finite sampling, the Gaussian noise model, in common with the Wright-Fisher model, exhibits frequency-dependent compound variance.

Ignoring the effect of the absorbing boundaries, the inherent variance of the Gaussian drift model is frequency-independent and increases linearly with time, as can be derived by applying the law of total expectation and

total variance:

$$Var_G^H(t_k) = \sigma^2 t_k \tag{E.4}$$

while the inherent expectation is constant

$$E_G^H(t_k) = E_G^H(t_{k-1}) = q(0) \tag{E.5}$$

A similar calculation for the Wright-Fisher drift model shows the expected frequency-dependent variance at each sampling time as:

$$E_{WF}^H[q(t_k)] = q(0) \tag{E.6}$$

$$Var_{WF}^H[q(t_k)] = q(0)(1-q(0))\left[1-(1-\tfrac{1}{2N})^{t_k}\right] \tag{E.7}$$

Applying once again the law of total expectation and variance for the sampling step we can obtain variances of the compound sampling problem, at a generation t_k, under the HMM chain associated with the likelihood function previously presented in Eq. E.1:

$$\begin{aligned} Var_G^S(t_k) &= E_G^S[q(t_k)](1-E_G^S(q(t_k)))\left[\frac{1}{C(t_k)}+\left(1-\frac{1}{C(t_k)}\right)\frac{1}{2N_{pool}}\right] \\ &+\left(1-\frac{1}{C(t_k)}\right)\left(1-\frac{1}{2N_{pool}}\right)Var_{WF}^H[q(t_k)] \\ &= q(0)(1-q(0))\left[\frac{1}{C(t_k)}+\left(1-\frac{1}{C(t_k)}\right)\frac{1}{2N_{pool}}\right] \\ &+\left(1-\frac{1}{C(t_k)}\right)\left(1-\frac{1}{2N_{pool}}\right)\sigma_G^2 t_k \end{aligned} \tag{E.8}$$

$$
\begin{aligned}
Var^S_{WF}(t_k) &= E^S_{WF}[q(t_k)](1-E^S_{WF}(q(t_k)))\left[\frac{1}{C(t_k)}+\Big(1-\frac{1}{C(t_k)}\Big)\frac{1}{2N_{pool}}\right] \\
&+\Big(1-\frac{1}{C(t_k)}\Big)\Big(1-\frac{1}{2N_{pool}}\Big)Var^H_{WF}[q(t_k)] \\
&= q(0)(1-q(0)) \\
&\times\left\{\left[\frac{1}{C(t_k)}+\Big(1-\frac{1}{C(t_k)}\Big)\frac{1}{2N_{pool}}\right]+\Big(1-\frac{1}{C(t_k)}\Big)\Big(1-\frac{1}{2N_{pool}}\Big)\left[1-(1-\frac{1}{2N})^{t_k}\right]\right\}
\end{aligned}
\tag{E.9}
$$

where $C(t_k)$ is the total read depth at a specific sampling generation and N_{pool} is the size of pool of individuals chosen for sequencing. In the case of the data used in the work presented here, 500 female flies were used for the pool [7] (see also Methods section in Main Text).

Given this calculation, a study was conducted of the extent to which the frequency-dependent variance observed in the data was reproduced by each model.

Considering the experimental data, observed allele frequencies were binned according to the predicted posterior means found for each locus and time-point. Plotting the variance of the allele frequency $q(t_{k+1})$ against the measure $q(t_k)(1-q(t_k))$ allowed us to verify the frequency dependence predicted by each drift model, either through the analytical derivations represented in Eqs. E.8 and E.9, or through the inferred posterior variances resulting from the combined forward-backward/predict-update HMM algorithm outlined above (Appendix E.1).

Given these measures, the mean squared error between the observed and inferred variances was calculated across the binned frequencies. Despite no

clear pattern being observed in these statistics for each replicate and chromosome, the Gaussian predicted variance calculated through the posterior outperforms the respective Wright-Fisher posterior model if the difference in mean squared error is summed across time-points and replicates (Figs. E.11 and E.12). With respect to the variance calculated by applying the analytical solutions represented in Eqs. E.8 and E.9, the opposite result is observed (Figs. E.11 and E.12).

Overall, the use of the posterior variances improves the inferred values of variance when the Gaussian drift model is used, which points to the advantage, in this case, of taking data into account in the HMM algorithm presented above. The same observation is not clearly verified for the Wright-Fisher model. This result contrasts with that reported in the main text where across all chromosomes and replicates the Wright-Fisher is the most representative. This further emphasizes the importance of higher distribution moments prevalent in the total likelihood approach and respective goodness-of-fit results. As stated in the Main Text, performance without trajectories reaching loss or fixation still favoured the Wright-Fisher model. Consistently with this, the results plotted in Figs. E.10, E.11, E.12 and E.13 were also found for trajectories that did not reach the frequency boundaries.

Curves predicted for the X chromosome are shown in Figure E.10 and the respective error is presented in Fig. E.11. Data for other chromosomes is shown in Fig. E.12 and E.13

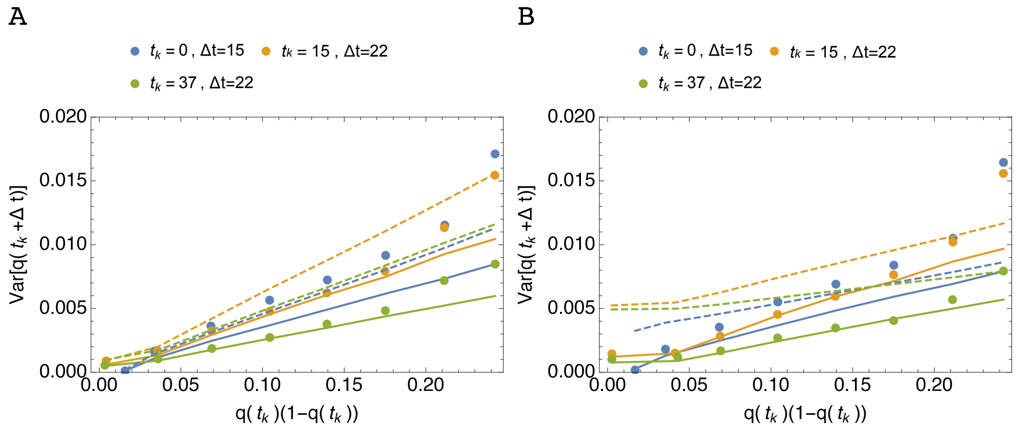

Figure E.10: **Estimates of compound distribution variance from Drosophila experimental evolution time-series [7] (chromosome X, replicate 1).** (A) Compound variance curves obtained with posterior means and variances (Full lines, $WFpost$) as well as with compound variance analytical expressions (Dashed, WF) (see Eqs. E.9 and E.8) for Wright-Fisher and (B) Gaussian drift models.

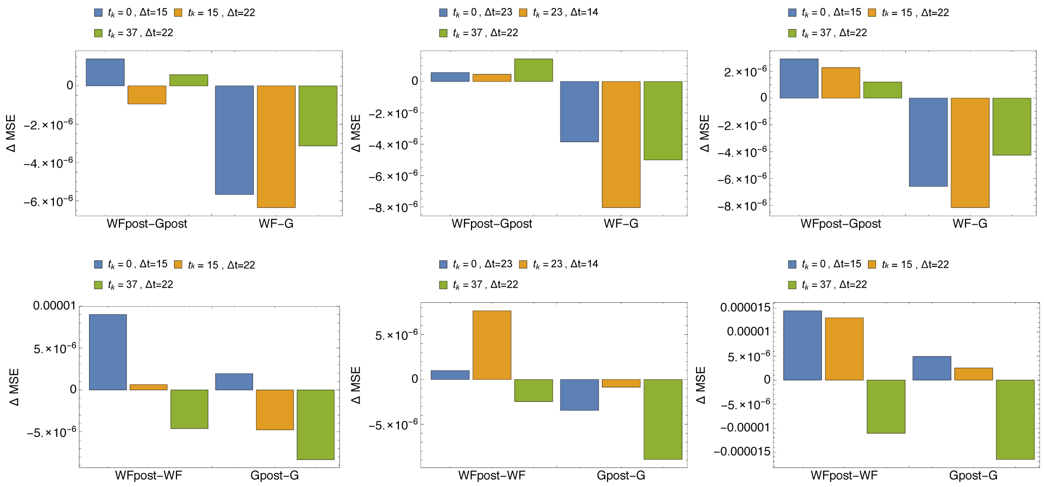

Figure E.11: **Difference in mean square error in the estimates of compound distribution variance from Drosophila experimental evolution time-series [7] for Wright-Fisher and Gaussian models (chromosome X).** WF_{post}, G_{post}: calculations with posterior variances. WF, G: Calculation with analytical solutions. From left to right: replicate 1, 2 and 3.

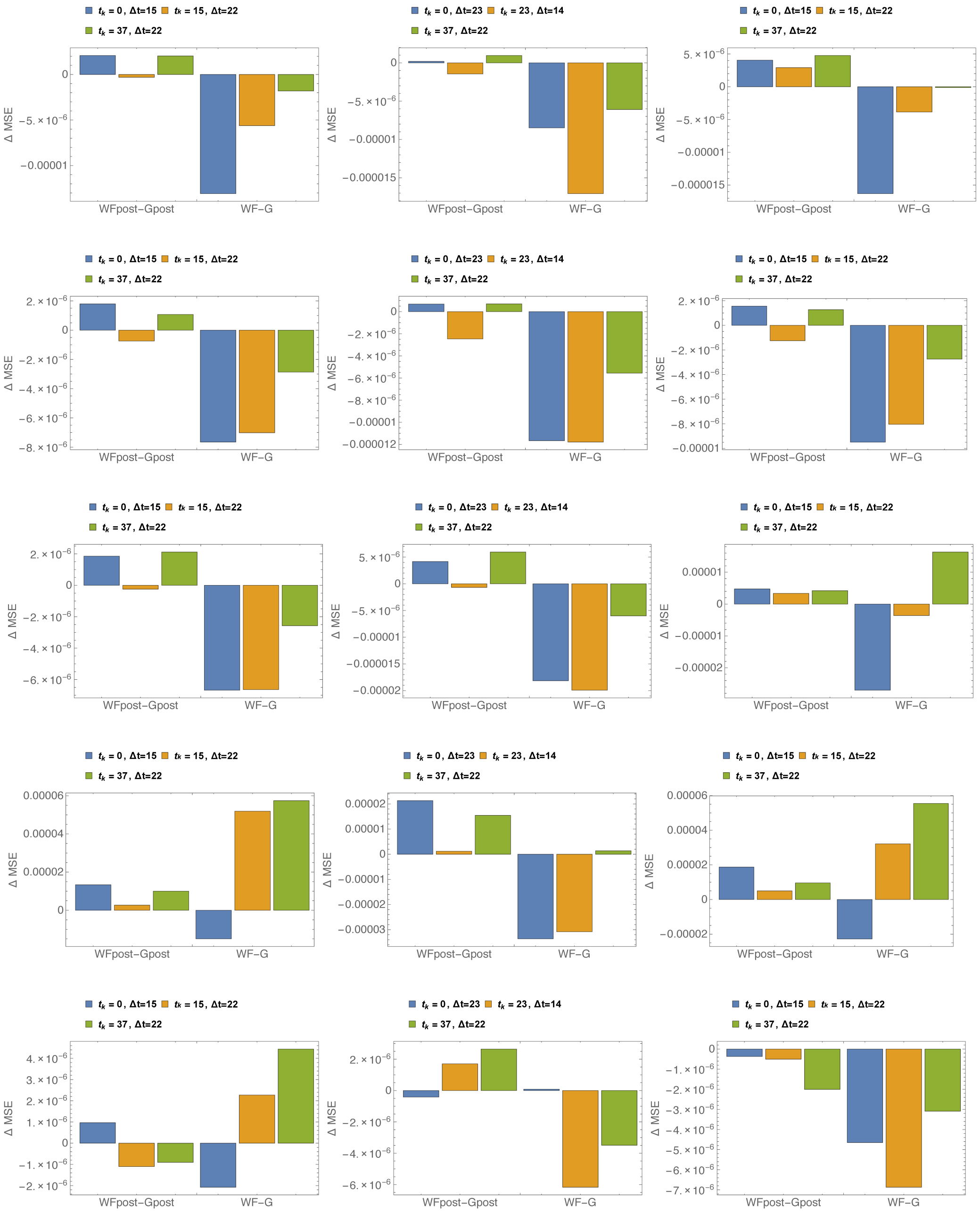

Figure E.12: **Difference in mean square error in the estimates of compound distribution variance from Drosophila experimental evolution time-series [7] for Wright-Fisher and Gaussian models.** WF_{post}, G_{post}: calculations with posterior variances. WF, G: Calculation with analytical solutions. From left to right: replicate 1, 2 and 3. From top to bottom: chromosome $2L, 2R, 3L, 3R, 4$.

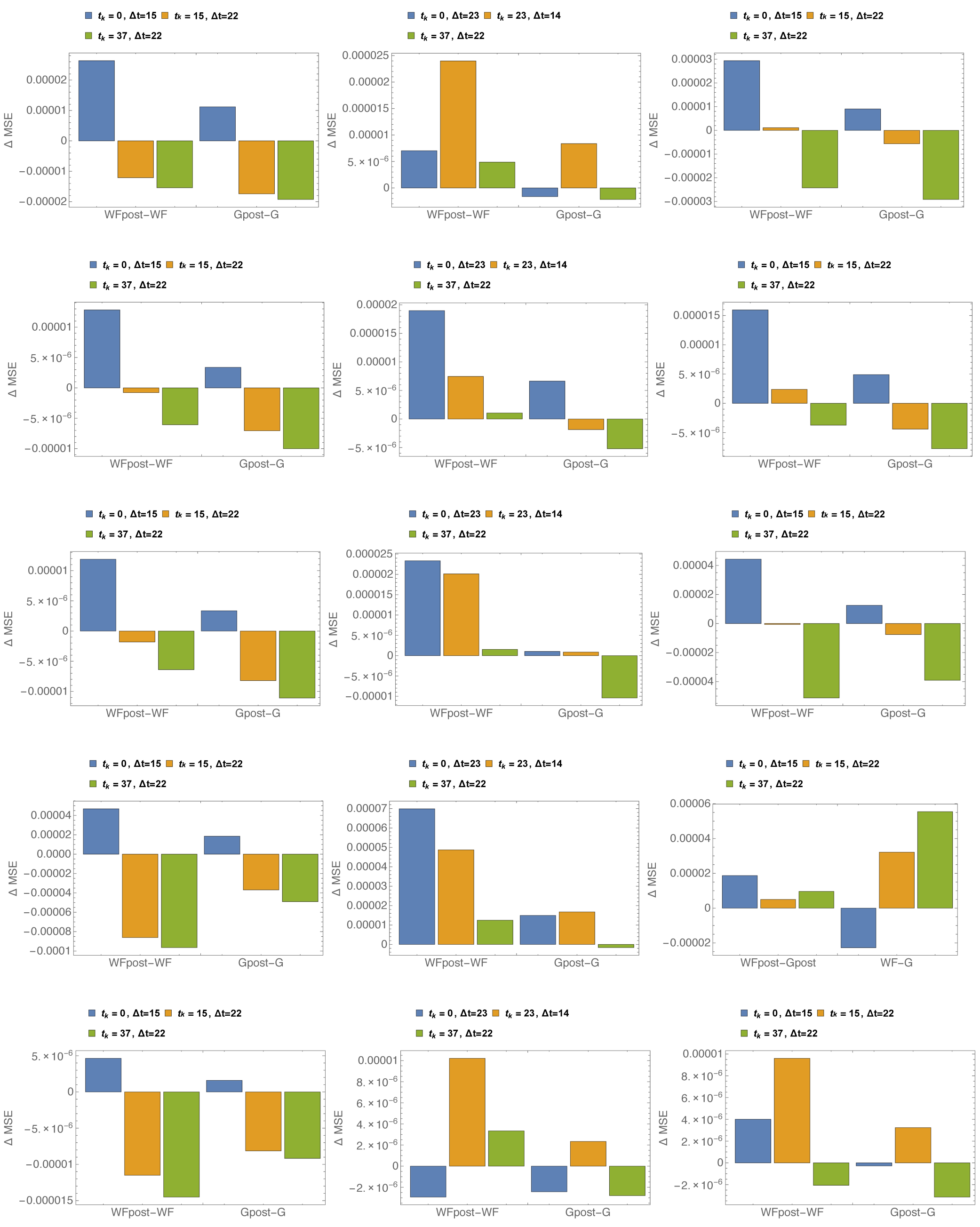

Figure E.13: **Difference in mean square error between estimates of compound distribution variance from Drosophila experimental evolution time-series [7] obtained with posterior variances and analytical solutions.** WF_{post}, G_{post}: calculations with posterior variances. WF, G: Calculation with analytical solutions. From left to right: replicate 1, 2 and 3. From top to bottom: chromosome $2L, 2R, 3L, 3R, 4$.

Appendix F. Frequency spectrum for experimental data

The probability density functions for each chromosome collected from [7] is shown in Figs. F.14. All instants when data was collected during the experiment are presented.

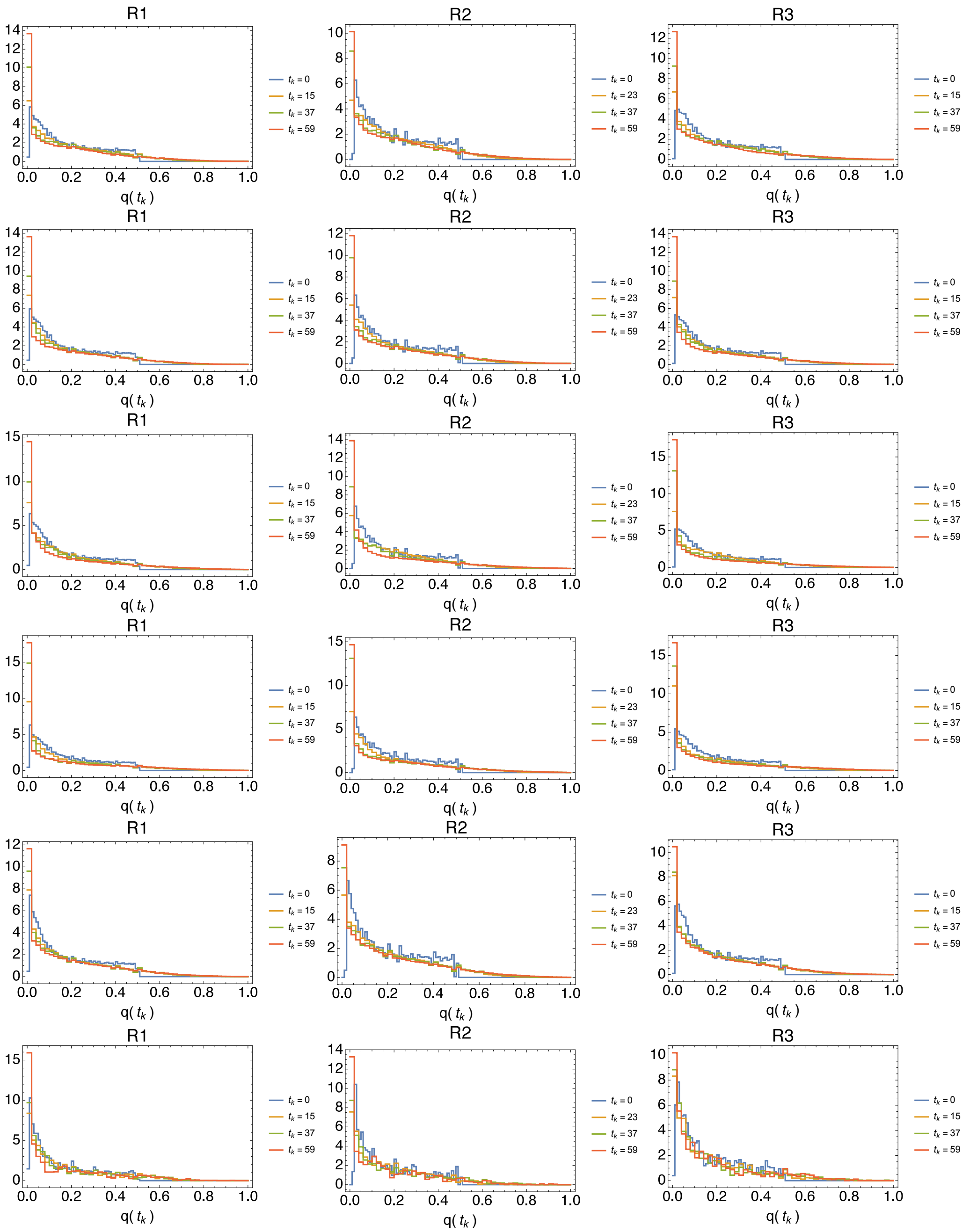

Figure F.14: **Frequency spectrum from Drosophila experimental evolution time-series measured by Pool-Seq [7].** From left to right: replicate $R1$, $R2$ and $R3$ (see Methods in Main Text). From top to bottom: chromosome $2L, 2R, 3L, 3R, X, 4$.

References

References

[1] R. Kofler, C. Schltterer, A guide for the design of evolve and resequencing studies, Molecular Biology and Evolution 31 (2) (2014) 474–483. doi:10.1093/molbev/mst221.

[2] J. Terhorst, C. Schltterer, Y. S. Song, Multi-locus analysis of genomic time series data from experimental evolution, PLoS Genet 11 (4) (2015) 1–29. doi:10.1371/journal.pgen.1005069.

[3] H. Topa, Á. Jónás, R. Kofler, C. Kosiol, A. Honkela, Gaussian process test for high-throughput sequencing time series: application to experimental evolution, Bioinformatics 31 (11) (2015) 1762–1770. doi:10.1093/bioinformatics/btv014.

[4] L. Zhao, M. Lascoux, D. Waxman, An informational transition in conditioned markov chains: Applied to genetics and evolution, Journal of Theoretical Biology 402 (2016) 158 – 170. doi:http://dx.doi.org/10.1016/j.jtbi.2016.04.021.

[5] H. Srensen, Parametric inference for diffusion processes observed at discrete points in time: A survey, International Statistical Review / Revue Internationale De Statistique 72 (3) (2004) 337–354.

[6] M. Lacerda, C. Seoighe, Population genetics inference for longitudinally-sampled mutants under strong selection, Genetics 198 (3) (2014) 1237–1250. doi:10.1534/genetics.114.167957.

[7] S. U. Franssen, V. Nolte, R. Tobler, C. Schlötterer, Patterns of linkage disequilibrium and long range hitchhiking in evolving experimental drosophila melanogaster populations, Molecular biology and evolution 32 (2) (2015) 495–509. doi:10.1093/molbev/msu320.

[8] I. M. Rouzine, A. Rodrigo, J. Coffin, Transition between stochastic evolution and deterministic evolution in the presence of selection: general theory and application to virology, Microbiology and molecular biology reviews 65 (1) (2001) 151–185.

[9] I. M. Rouzine, L. S. Weinberger, The quantitative theory of within-host viral evolution, Journal of Statistical Mechanics: Theory and Experiment 2013 (01) (2013) P01009.

[10] G. Martin, A. Lambert, A simple, semi-deterministic approximation to the distribution of selective sweeps in large populations, Theoretical population biology 101 (2015) 40–46.

[11] H. Li, W. Stephan, Inferring the demographic history and rate of adaptive substitution in drosophila, PLOS Genetics 2 (10) (2006) 1–10. doi:10.1371/journal.pgen.0020166.
